# Supplementary material for: Strain-Insensitive Elastic Surface Electromyographic (sEMG) Electrode for Efficient Recognition of Exercise Intensities
Source: Micromachines (Basel). 2020 Feb 25;11(3):239. doi: 10.3390/mi11030239 (PMC7143104; doi:10.3390/mi11030239)
Supplement: Supplementary file 1 [file micromachines-11-00239-s001.pdf]

micromachines

Supplementary Materials

Strain-Insensitive Elastic Surface Electromyographic (sEMG) Electrode for Efficient Recognition of Exercise Intensities

Daxiu Tang [1,2,3], Zhe Yu [2,3,4], Yong He [2,3], Waqas Asghar [2,3,5], Ya-Nan Zheng [2,3,4], Fali Li [2,3,4], Changcheng Shi [2,3], Roozbeh Zarei [2,3,6], Yiwei Liu [2,3,4], Jie Shang [2,3,4,*], Xiang Liu [1,*] and Run-Wei Li [2,3,4,*]

1 Faculty of Materials Science and Engineering, Kunming University of Science and Technology, Kunming 650093, China
2 CAS Key Laboratory of Magnetic Materials and Devices, Ningbo Institute of Materials Technology and Engineering, Chinese Academy of Sciences, Ningbo 315201, China
3 Zhejiang Province Key Laboratory of Magnetic Materials and Application Technology, Ningbo Institute of Materials Technology and Engineering, Chinese Academy of Sciences, Ningbo 315201, China
4 Center of Materials Science and Optoelectronics Engineering, University of Chinese Academy of Sciences, Beijing 100049, China
5 Department of Mechanical Engineering, University of Engineering and Technology Taxila, Taxila 47050, Pakistan
6 Swinburne Data Science Research Institute, Swinburne University of Technology, Melbourne VIC 3122, Australia
* Correspondence: shangjie@nimte.ac.cn (J.S.); lxjim@126.com (X.L.); runweili@nimte.ac.cn (R.L.)

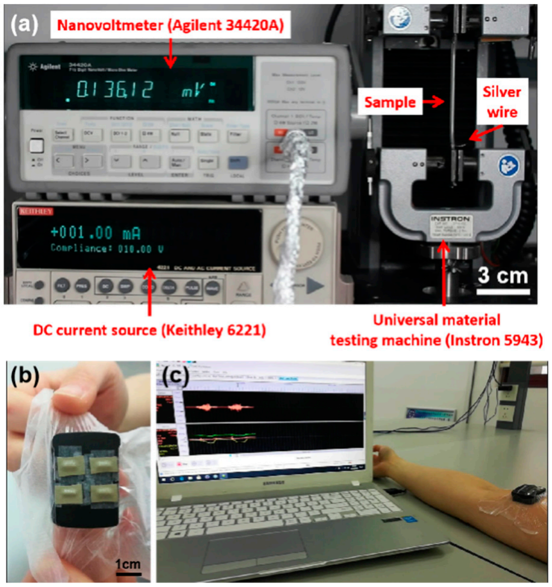

Figure S1. Photos of (**a**) electromechanical measurement, (**b**) the connection between electrode and surface electromyography (sEMG) device, and (**c**) sEMG signal collection.

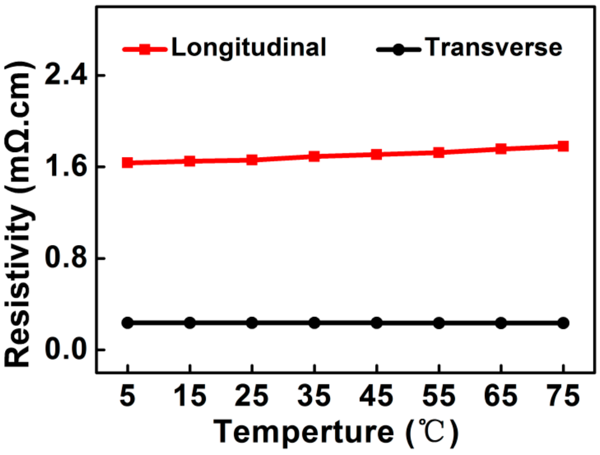

Figure S2. Resistivity variation of electrode at different temperatures.

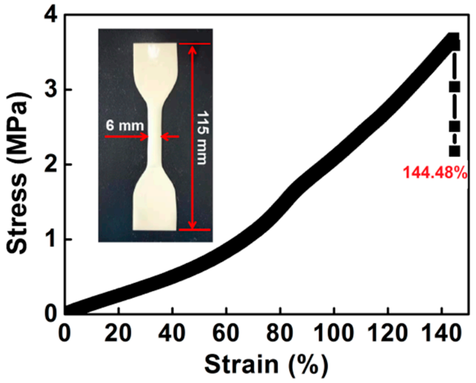

Figure S3. Tensile stress–strain curve of our sEMG electrode.

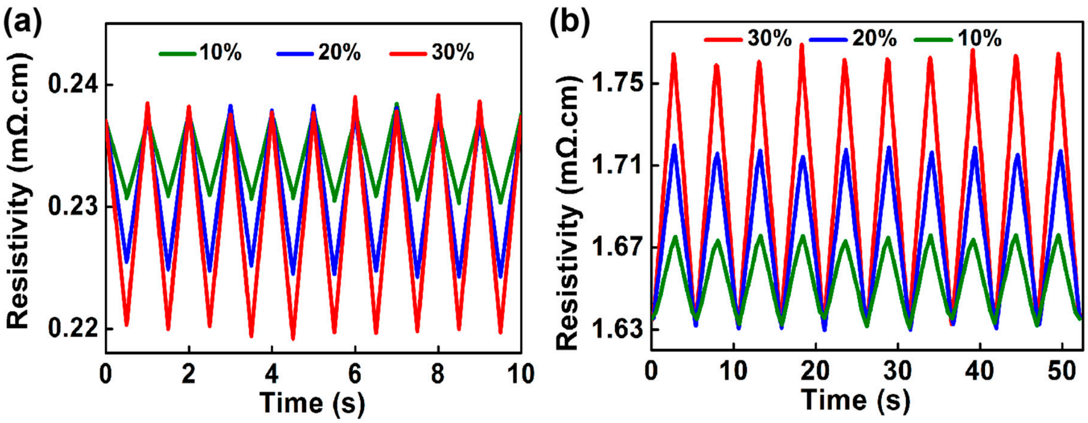

Figure S4. Resistivity change of three-layered sEMG electrode in different directions with the repeated loading and unloading of various strains: (**a**) transverse resistivity and (**b**) longitudinal resistivity.

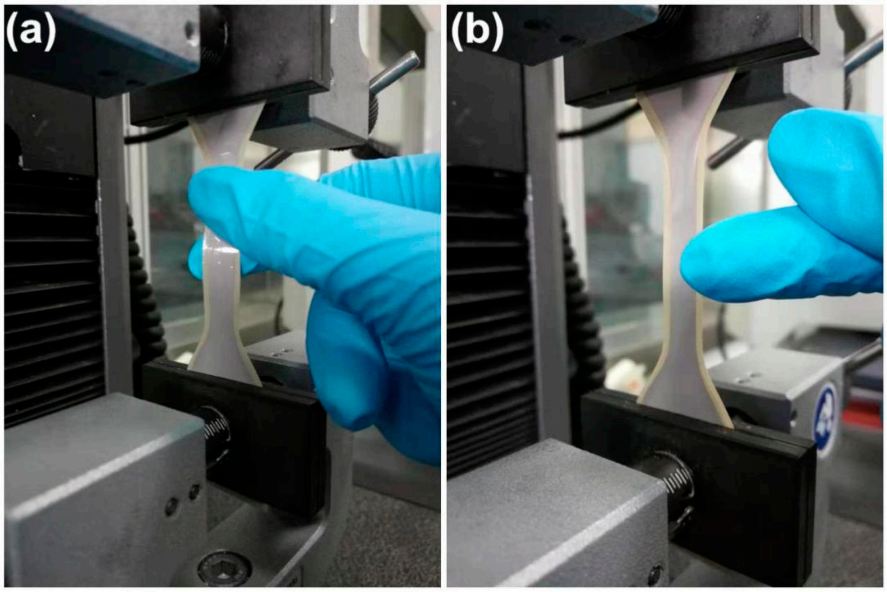

Figure S5. Verification of LM(LM means galinstan) leakage. (**a**) Repeated stretching of sEMG electrode for 100 times (at 30% stretching strain). (**b**) After stretching and rubbing no sign of LM is found, which indicates the high skin-friendliness of the electrode.

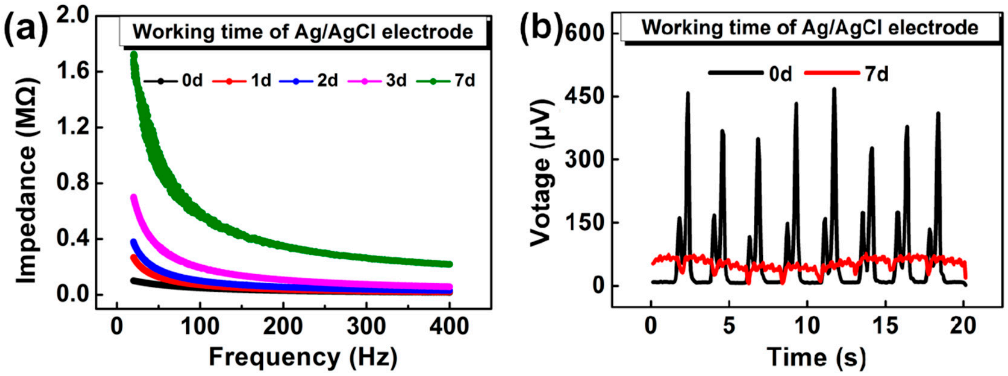

Figure S6. Stability test of Ag/AgCl electrode. (**a**) Time-dependent impedance increase of Ag/AgCl electrode. (**b**) sEMG signal of fresh Ag/AgCl electrode and after the passage of 7 days.

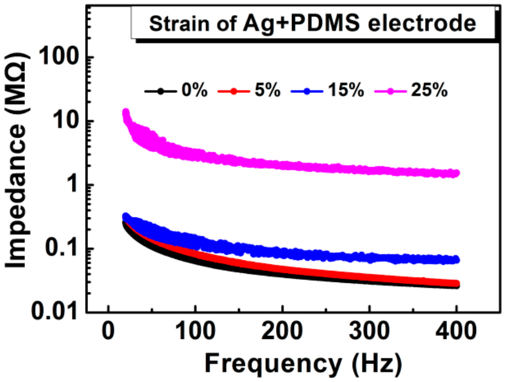

Figure S7. Impedance change of traditional Ag + polydimethylsiloxane (PDMS) electrode (1:1 mass ratio) under different stretching strains.

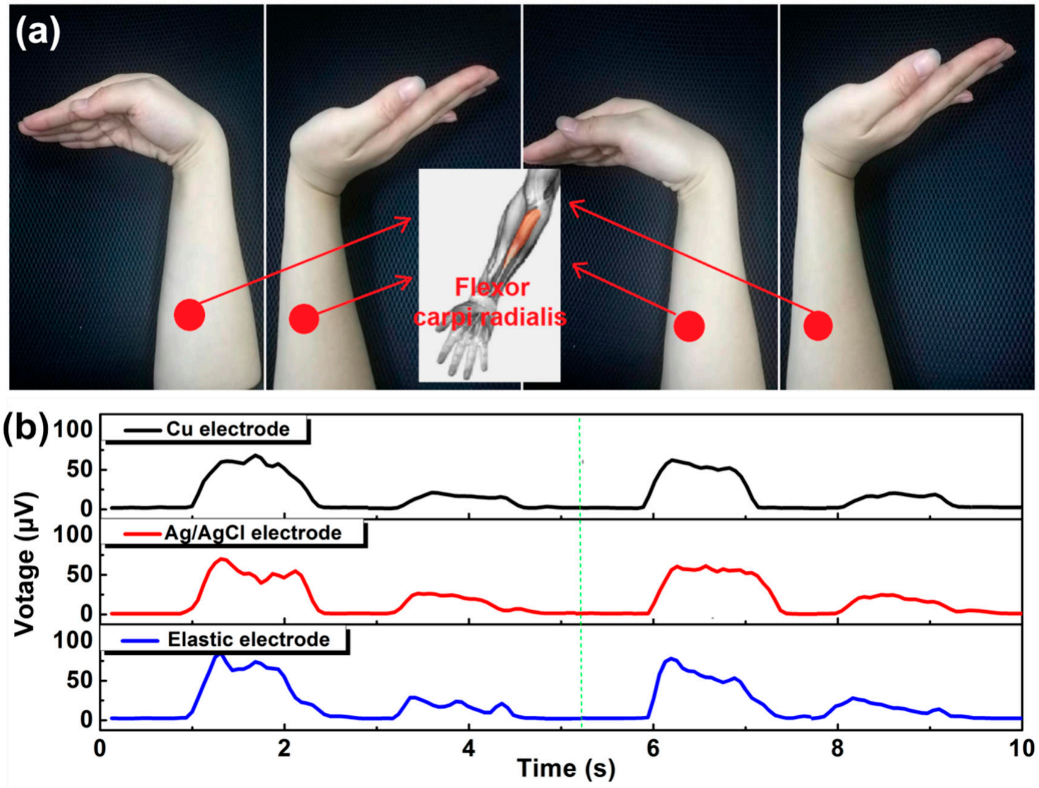

Figure S8. sEMG signal test for recording various gestures of a human hand. (**a**) Various gestures of a human hand. (**b**) Resultant sEMG signals.

© 2020 by the authors. Submitted for possible open access publication under the terms and conditions of the Creative Commons Attribution (CC BY) license (http://creativecommons.org/licenses/by/4.0/).
